# ADC Histograms from Routine DWI for Longitudinal Studies in Cerebral Small Vessel Disease: A Field Study in CADASIL

**DOI:** 10.1371/journal.pone.0097173

**Published:** 2014-05-12

**Authors:** Bence Gunda, Raphael Porcher, Marco Duering, Jean-Pierre Guichard, Jerome Mawet, Eric Jouvent, Martin Dichgans, Hugues Chabriat

**Affiliations:** 1 Department of Neurology, Semmelweis University, Budapest, Hungary; 2 Department of Neurology, CHU Lariboisière, DHU NeuroVasc, APHP and Université Paris Denis-Diderot, Paris, France; 3 Department of Neuroradiology, CHU Lariboisière, DHU NeuroVasc, APHP and Université Paris Denis-Diderot, Paris, France; 4 INSERM UMR 1161, Paris, France; 5 Université Paris Descartes, Paris, France; 6 Institute for Stroke and Dementia Research, Klinikum der Universität München, Ludwig-Maximilians-University, Munich, Germany; 7 Munich Cluster for Systems Neurology, Munich, Germany; Aarhus University, Denmark

## Abstract

Diffusion tensor imaging (DTI) histogram metrics are correlated with clinical parameters in cerebral small vessel diseases (cSVD). Whether ADC histogram parameters derived from simple diffusion weighted imaging (DWI) can provide relevant markers for long term studies of cSVD remains unknown. CADASIL patients were evaluated by DWI and DTI in a large cohort study overa6-year period. ADC histogram parameters were compared to those derived from mean diffusivity (MD) histograms in 280 patients using intra-class correlation and Bland-Altman plots. Impact of image corrections applied to ADC maps was assessed and a mixed effect model was used for analyzing the effects of scanner upgrades. The results showed that ADC histogram parameters are strongly correlated to MD histogram parameters and that image corrections have only limited influence on these results. Unexpectedly, scanner upgrades were found to have major effects on diffusion measures with DWI or DTI that can be even larger than those related to patients’ characteristics. These data support that ADC histograms from daily used DWI can provide relevant parameters for assessing cSVD, but the variability related to scanner upgrades as regularly performed in clinical centers should be determined precisely for longitudinal and multicentric studies using diffusion MRI in cSVD.

## Introduction

CADASIL (Cerebral Autosomal Dominant Arteriopathy with Subcortical Infarcts and Leukoencephalopathy) is the most frequent hereditary cerebral small vessel disease (cSVD) characterized by recurrent stroke and early cognitive decline affecting middle-aged adults. It is considered as a unique model to investigate the pathophysiology of subcortical ischemic vascular dementia related to cSVD.[1]Conventional magnetic resonance imaging (MRI) provides key information for diagnosis of the disease. FLAIR or T2-weighted images show diffuse white matter signal abnormalities in all symptomatic but also in asymptomatic CADASIL patients[2]. T1-weighted images often show lacunar infarctions accumulating progressively with the progression of the disease in two thirds of patients[2].

Unlike conventional T1 and T2-weighted MRI sequences, diffusion MRI can probe the microstructural integrity of cerebral tissue and was shown to be highly sensitive to cerebral tissue changes in cSVD[3]. Important changes of diffusion tensor imaging (DTI) metrics (mean diffusivity –MD and fractional anisotropy –FA) have been reported both inside and outside areas of increased signal on T2-weighted or FLAIR images in various white-matter disorders.[4]–[9]In conditions with diffuse tissue lesions such as hypertension related cSVD or CADASIL, a quantitative approach based on whole brain histograms of diffusion was found to reflect the overall disease severity and various DTI histogram parameters (mean value, median value, peak location, peak height, kurtosis, skewness) have been reported to correlate with clinical scores both in cross-sectional and longitudinal studies.[10]–[20]Some DTI metrics were even found more sensitive than clinical scales in detecting the disease progression over time[11], [15], [21]. In CADASIL, mean value of MD histograms obtained over the whole brain has been previously found to increase before any significant clinical change during follow up and to predict disease progression [21], [22]. DTI measures were then proposed as potential adjunct outcome measures for future therapeutic trials in cSVD[10], [21]–[24]. However, the effects of variations in sequences or scanners on diffusion measures, which are of crucial importance for multicentric and longitudinal studies, have only been evaluated in limited samples and mainly in healthy volunteers[25]–[28]. In addition, for large scale multicentric studies, a very simple and highly reproducible measure derived from diffusion histograms appears strongly needed. Apparent diffusion coefficient (ADC) histograms obtained from measures in 3 gradient directions (x, y and z directions) over the whole brain without the use of any operator-dependent and time-consuming post-processing as daily performed in stroke patients may represent an alternative approach to DTI-metrics based on longer acquisitions and more sophisticated calculations. The main advantage of using ADC histograms would be its wide availability, simplicity and rapidity making it also suitable for routine clinical use.

The aims of the present study were: 1) to evaluate whether very simple parameters derived from ADC histograms can be used similarly to DTI histogram parameters to assess the severity of cSVD, 2) to assess the magnitude of the effect of scanner upgrades or changes that necessarily occur in clinical studies over a large time scale- as compared to the effect of clinical scores, age and sex on ADC histogram parameters. For this purpose, clinical and MRI data from a large cohort of CADASIL patients evaluated in two different clinical centers and over a long time period were analyzed.

## Methods

### Subjects

Data from 771 MRI scans performed in 348 CADASIL patients having a typical mutation of the Notch3 gene were used for analysis in this study. Patients were recruited from 2006 to 2012ina large cohort study performed in two referral centers (Lariboisière Hospital in Paris and Institute for Stroke and Dementia Research in Munich). The detailed design of this study has been previously reported elsewhere.[29]The study was approved by an independentethics committee at both centres (Paris: Ethics Committee of Saint Louis Hospital, reference no. P020921-AOR02001; Munich: Ethikkommission der Med. Fakultät der LMU München), all patients gave a written informed consent to participate.

### Clinical evaluation

All subjects underwent a detailed neurological examination during the 2 hours before MRI, including the evaluation of the NIHSS, modified Rankin Scale, Barthel Index and Mattis Dementia Rating Scale. Patients had follow-up examinations with an interval of 18 months over a period of 3 years.

### MRI

MRI scans were obtained with 1.5T scanners (Vision[Siemens]in Munich and Signa [General Electric Medical Systems] in Paris) with repeated software and hardware (coil, channel RF and other technical element) updates (GE Signa 08, 09, 11, 11 new, 12) as proposed by the manufacturer in all clinical centers during the study period. Diffusion weighted imagingwas performed in all patients (Siemens: TR/TE 5100/137 ms, slice thickness 5 mm, interslice gap 1.5 mm, 128×128; b-value = 1000; General Electric: TR/TE 8200/83 ms, slice thickness5.5 mm, interslice gap 1.5 mm, 128×128; b value = 1000 s/mm^2^). To obtain ADC maps, DWI scans were acquired in the X, Y, and Z directions and then averaged to make ADC measurements largely independent of the effects of anisotropic diffusion. Apparent diffusion coefficient values were then calculated for each voxel to generate ADC_xyz_ maps. In a subset of patients (n = 280) diffusion tensor imaging was also performed using a unique and optimized protocol on GE Signa in 23 directions (TR: 7500, TE: 98.8 ms, EC: 1/1, bandwidth: 91 Khz, slice thickness: 5.5 mm, inter slice gap 1.5 mm, 23 slices, 128×128, b value = 700 s/mm^2^in 23 directions). Eigen-vectors were obtained from all 23 directions for each voxel and eigen values were used for calculation of mean diffusivity (MD  =  Trace/23). Data obtained using other DTI sequences that were performed during the study were not included in this analysis.

### Image analysis

MD and ADC were first calculated over the whole volume of the brain. In the present study, all ADC histogram parameters obtained using DWI were compared to mean diffusivity (MD) histogram parameters obtained using DTI at the same time and considered as the reference method. MD histograms were generated after removal of voxels containing CSF using a cutoff value of diffusion at 18×10^−4^ mm^2^/s. This cutoff level was chosen after careful visual assessment of the effect of different thresholds.

In search for the simplest measure of diffusion, we evaluated the effects of different image corrections usually requested for DTI measures (cerebrospinal fluid (CSF) suppression, automatic and manual artifacts removal) on ADC histogram parameters. ADC histograms were generated before and after applying 3 types of image correction on crude ADC maps; 1) suppression of CSF with a threshold value at 18×10^−4^ mm^2^/s; 2) automatic removal of the top and bottom three slices containing the most important artifacts or peripheral CSF; 3) manual removal of artifacts at the bone-air interface by an experienced neurologist (JM).Histograms were obtained using a bin width of 0.1×10^−4^ mm^2^/s and normalized over the number of voxels to correct for individual differences in brain size. The mean value, peak location and peak height of diffusion histograms were used for analysis. Because height, kurtosis and skewness -parameters that represent the histogram curve- were found to be highly correlated to each other, we chose to only include height in the final analysis.

### Statistical methods

All analyses were made taking into account the 5 hardware upgrades performed on the GE scanner during the study period (Signa 08, 09, 11, 11 new and 12+). The relationship between MD and ADC histogram parameters was evaluated by the correlation coefficient, linear regression, intra-class correlation and Bland-Altman plots. A similar methodology was used to compare corrected and non-corrected ADC histograms. Finally, a mixed-effects model was used to assess the magnitude of the effect of scanner upgrades versus clinical scores on ADC histogram parameters. In these regression models adjusted for age and gender, a random scanner upgrade effect was used, to model an expected magnitude of scanner updates in general (standard error of the random effect), that can be compared to the (fixed) effect of clinical scores on ADC histogram parameters. All tests were two-sided and *p*-values <0.05 were considered as indicating significant association. Analyses were performed using the R statistical software version 2.15.0.

## Results

### Comparison of ADC histograms to the reference method according to scanner upgrades

**Figure 1:**
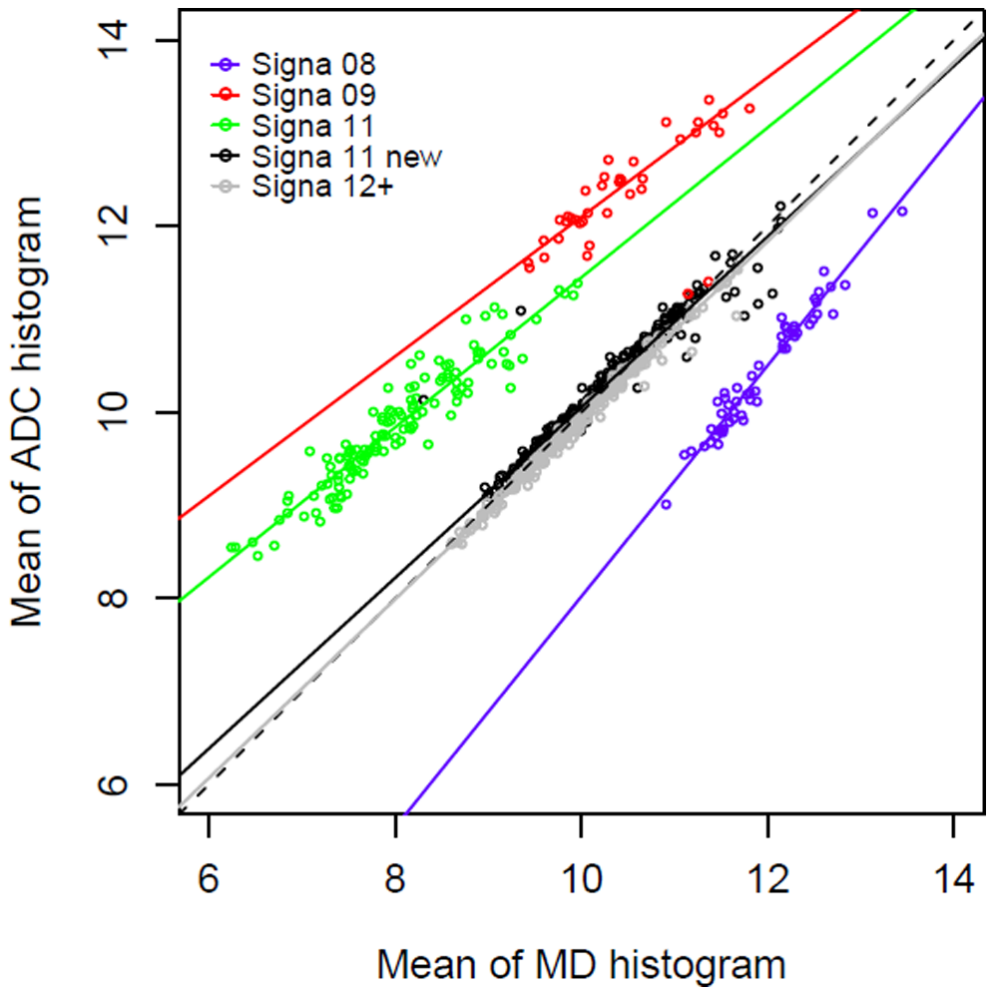
Linear regression analysis of mean value derived from ADC histograms vs mean MD values obtained with DTI after CSF removal (x 10^−4^ mm^2^/s) on the GE scanner. The graph shows the large effects of different scanner versions (software and hardware updates). If the ADC and MD measures appear well correlated overall, the graph illustrates that they clearly depend on the scanner version and are not concordant since all regression lines significantly differ from the perfect concordance line (dashed line).

Correlation coefficients between ADC and MD histogram parameters (as the reference method) were found high (all r> 0.75 with p values less than 0.0001) but to differ somewhat according to the scanner upgrade (Figure 1 and Table 1). Intra-class correlation coefficients (Table 2), corresponding to the ratio of the inter-patient variance to the overall variance of measurements, were found high independently of the scanner software version for the mean and height values of ADC histograms obtained after removal of CSF. Without CSF removal, the results appeared more variable according to the scanner version. The tightest correspondence with the reference method was observed for the mean value of ADC histograms.

**Table 1:** Correlation coefficients between ADC and MD histogram parameters (n denotes the number of scans); all p-values are less than 0.0001

MRI/software	Mean w/o CSF	Mean w CSF	Height w/o CSF	Height w CSF	Peak
Signa 08 (*n* = 56)	0.975	0.989	0.957	0.972	0.889
Signa 09 (*n* = 38)	0.681 *	0.879	0.877	0.884	0.252 **
Signa 11 (*n* = 129)	0.946	0.952	0.971	0.978	0.888
Signa 11 new (*n* = 276)	0.959	0.977	0.989	0.991	0.790
Signa 12+ (*n* = 204)	0.993	0.994	0.990	0.993	0.926

Values of 0.935* and 0.779** after removal of two outliers.

**Table 2:** Intra-class correlation coefficient between ADC and MD histogram parameters (n denotes the number of scans).

MRI/software	Mean w/o CSF	Mean w CSF	Height w/o CSF	Height w CSF	Peak
Signa 08 (n = 56)	0.691	0.777	0.836	0.855	0.477
Signa 09 (n = 38)	0.698*	0.850	0.789	0.826	0.202**
Signa 11 (n = 129)	0.919	0.770	0.811	0.835	0.797
Signa 11 new (n = 276)	0.944	0.964	0.927	0.939	0.722
Signa 12+ (n = 204)	0.990	0.986	0.986	0.990	0.904

Values of 0.833* and of 0.719** after removal of two outliers.

### Effects of imaging data corrections on ADC histogram parameters according to scanner upgrades

**Figure 2:**
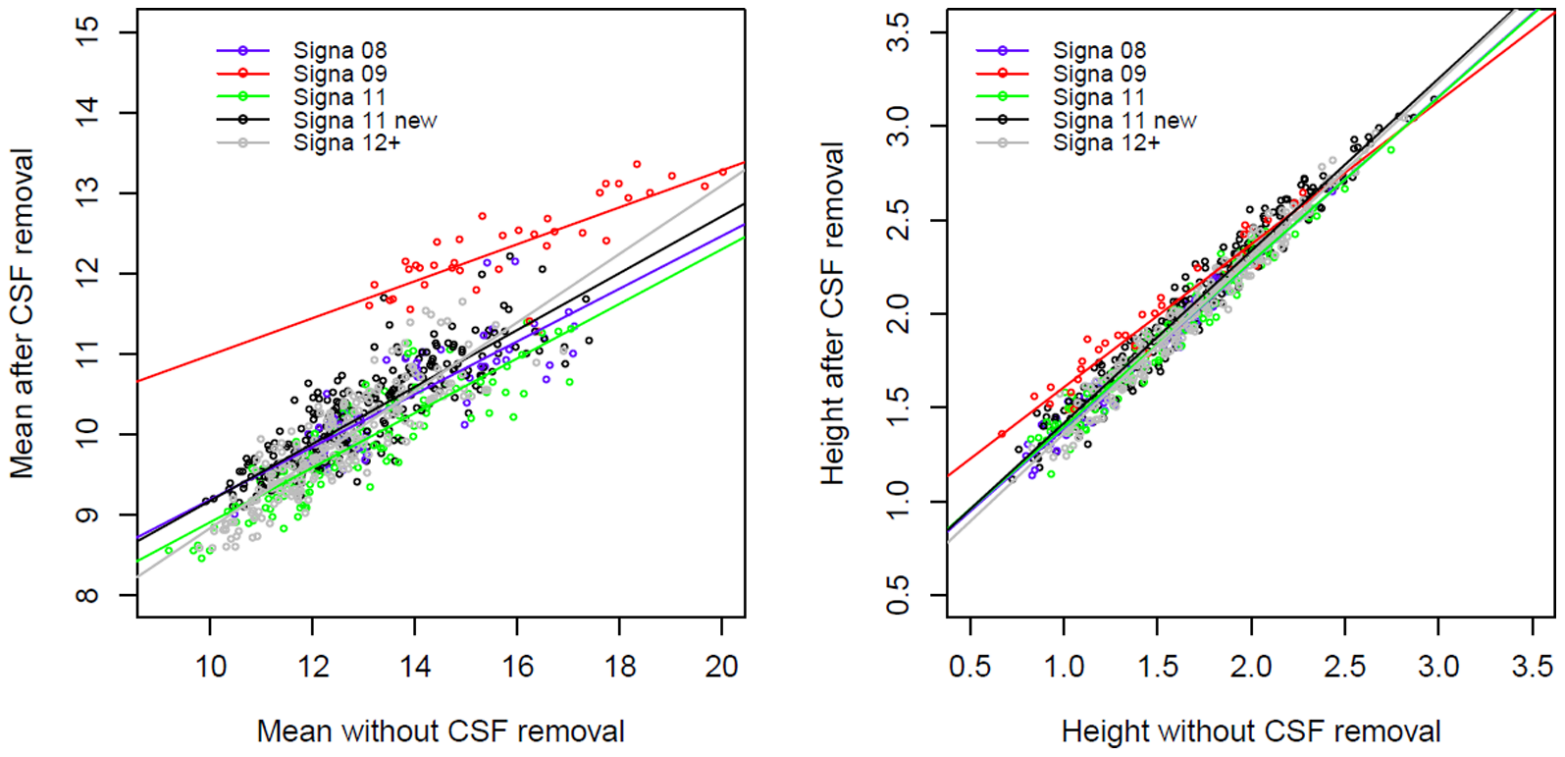
Linear regression analysis of mean value of ADC with and without CSF removal, showing the effects of different scanner versions.

**Figure 3:**
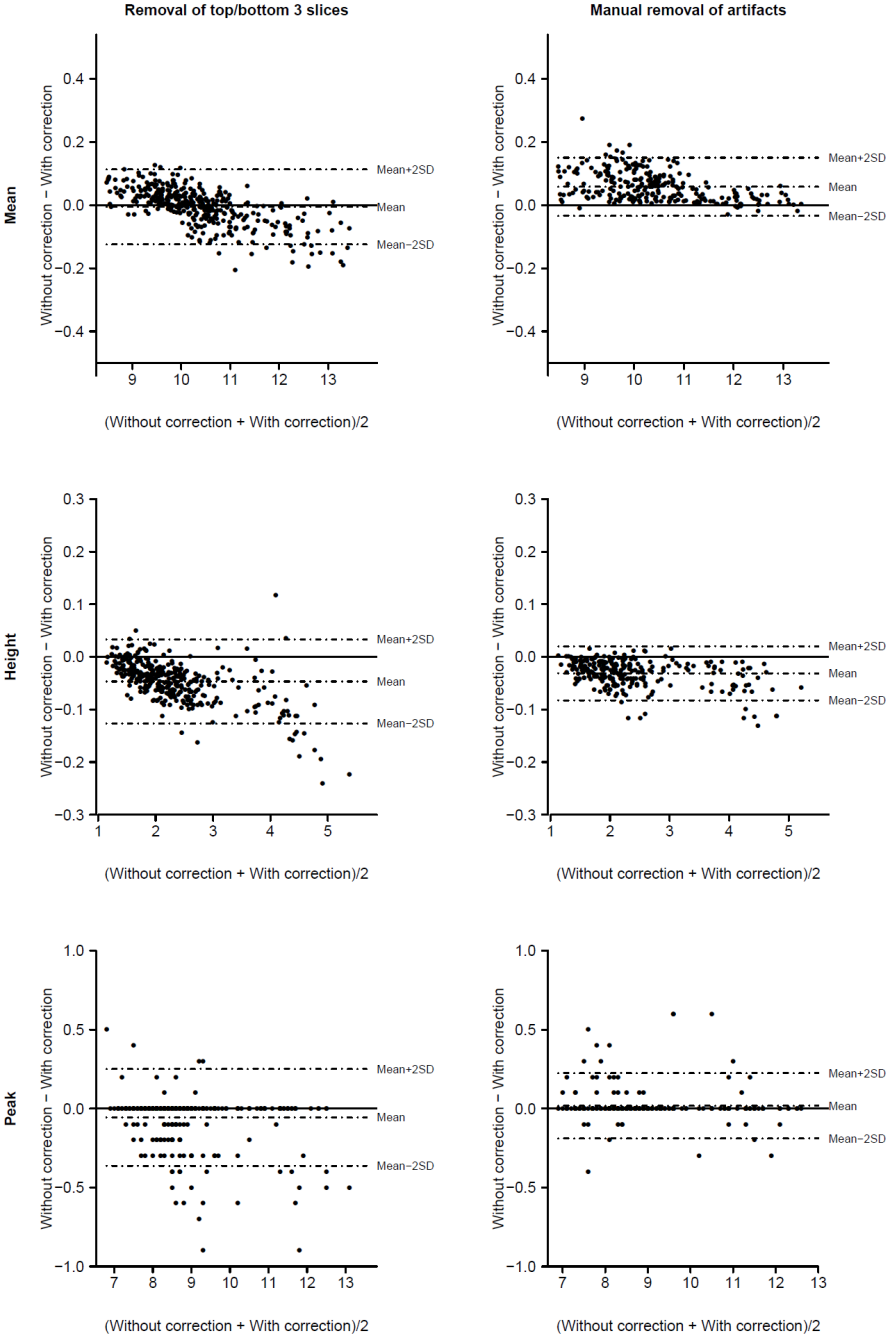
Bland–Altman plots for parameters derived from ADC histograms with and without application of different image corrections.

The intra-class correlation coefficients between corrected and non-corrected ADC histogram parameters are presented in Table 3. Intra-class correlation of parameters with/without CSF removal was found lower for the mean value than for the height of ADC histograms. Peak location was unaffected by CSF removal (as expected and not reported here). Plotting parameters with versus without CSF removal revealed a clear scanner version effect for the mean value, but also for the height, although less marked (Figure 2). Histogram parameters with/without removal of artifacts obtained either automatically (top-bottom three slices) or manually (bone-air artifacts) were almost perfectly correlated, in particular for the mean and height values of ADC histograms. Moreover, no strong scanner software version effect was found, thus data were analyzed all together by Bland–Altman plots (Figure 3). These plots showed that narrow limits of agreement were obtained for all parameters, showing small or even negligible differences between parameters with and without correction. However, automatic removal of top/bottom three slices yield to a drift of mean and height values. Smaller values of parameters were slightly underestimated and larger values slightly overestimated. No such drift was observed with manual removal of bone-air artifacts, but a small downward bias was observed for the mean and a small upward bias for the height.

**Table 3:** Intra-class correlation between ADC histogram parameters obtained with and without different image corrections (n denotes the number of scans) (only baseline scans were used in these analyses).

	Removal of CSF			Removal of top/bottom 3 slices				Manual removal of artifacts			
MRI/software	*n*	Mean	Height	*n*	Mean	Height	Peak	*n*	Mean	Height	Peak
Signa 08	56	0.716	0.964	60	0.997	0.997	0.989	60	0.990	0.989	0.962
Signa 09	38	0.678	0.920	38	0.999	0.998	0.989	38	0.990	0.983	0.922
Signa 11	129	0.723	0.963	43	0.995	0.996	0.988	43	0.997	0.993	0.972
Signa 11 new	276	0.728	0.974	20	0.993	0.997	0.899	81	0.991	0.996	0.947
Signa 12+	204	0.758	0.978	40	0.998	0.998	0.969	78	0.993	0.995	0.962
Siemens				48	0.997	0.995	0.977	48	0.992	0.978	0.960

All p-values are less than 0.0001

### Evaluation and impact of scanner upgrades

**Figure 4:**
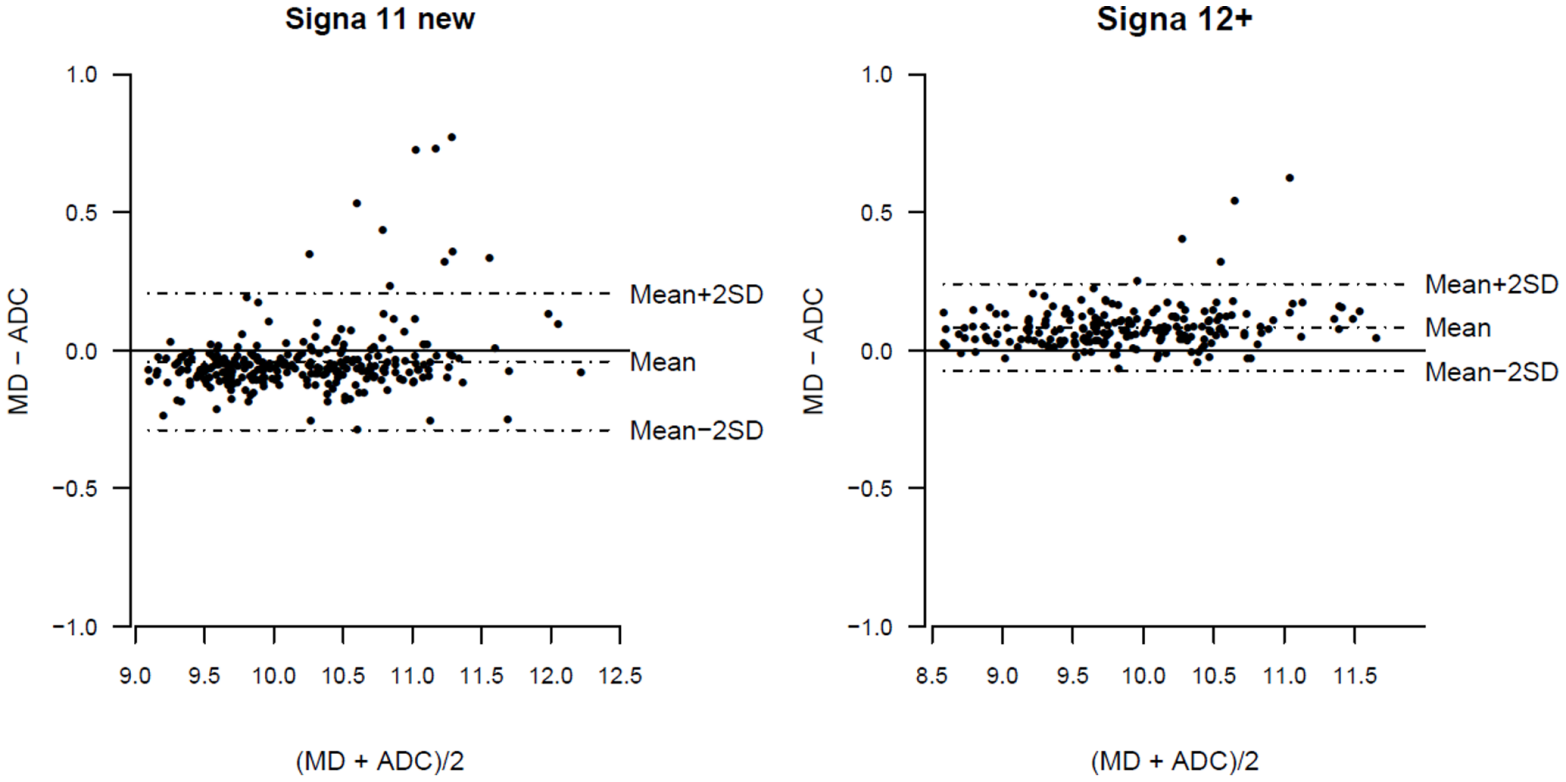
Bland–Altman plots for the mean value of ADC vs MD histograms after CSF removal on GE scanners with upgrade Signa 11 new and Signa 12+.

A strong effect of scanner upgrade was detected on all diffusion parameters (measured both on DTI and DWI). This is illustrated on Figure 1 for the mean values of whole brain histograms after CSF removal. The latest scanner software upgrades Signa 11 new and Signa 12 were the only ones for which all parameters measured by DTI (MD) or DWI (ADC) histograms were found highly concordant, with intraclass correlation coefficients above 0.9 (Table 2).On the contrary, the other scanner upgrades showed a smaller concordance, and the mean value and peak of the histogram were even very poorly correlated with Signa 09 version. Finally, Bland–Altman plots were also obtained for Signa 11 new and 12 versions of scanner since for the other versions, clear biases and deviations were expected. The corresponding plots are presented in Figure 4 showing small limits of agreement (mean difference ± 2 SD) between the mean value of ADC and MD histograms, after CSF removal in all cases. Some evidence of downward bias for mean ADC measures was found for Signa 11 new, with a mean difference of −0.04 (95%CI −0.06 to −0.03) and of upward bias with Signa 12, with which the mean difference was 0.08 (95%CI 0.07 to 0.09). Despite these differences, limits of agreement were −0.3 and 0.2 for Signa 11 new and −0.1 and 0.2 for Signa 12, which are small enough as compared to the measurements of DTI and DWI which span over the range of 8.5 to 12.5, with an average of about 10.

The effect of clinical scores, age and sex as compared to the random MRI scanner effect on different ADC histogram parameters evaluated in a mixed-effects model is presented in Table 4. The results showed that, globally, the standard deviation of the random scanner effect was larger than the regression coefficients of fixed effects of clinical scores, age or sex on ADC histogram parameters.

**Table 4:** Effects of clinical scores, age and sex (represented by their regression coefficients) compared to the random scanner effect (represented by its standard deviation) on different ADC histogram parameters.

Clinical score	Rankin		NIHSS		Barthel at 100	
	Coefficient (SE)	P	Coefficient (SE)	P	Coefficient (SE)	P
ADC mean						
Score	0.55 (0.04)	<0.0001	0.18 (0.02)	<0.0001	−1.44 (0.15)	<0.0001
Age	0.051 (0.006)	<0.0001	0.073 (0.006)	<0.0001	0.061 (0.006)	<0.0001
Male gender	0.37 (0.11)	0.002	0.40 (0.13)	0.002	0.49 (0.12)	<0.0001
Random scanner effect						
SD of random effect (SE)	1.01 (0.06)		0.98 (0.07)		1.03 (0.05)	
ADC mean without CSF						
Score	0.19 (0.02)	<0.0001	0.053 (0.010)	<0.0001	−0.49 (0.07)	<0.0001
Age	0.022 (0.003)	<0.0001	0.030 (0.003)	<0.0001	0.025 (0.003)	<0.0001
Male gender	0.097 (0.052)	0.065	0.11 (0.06)	0.049	0.14 (0.05)	0.013
Random scanner effect						
SD of random effect (SE)	0.76 (0.06)		0.76 (0.06)		0.76 (0.06)	
ADC peak location						
Score	0.13 (0.02)	<0.0001	0.038 (0.011)	<0.0001	−0.35 (0.08)	<0.0001
Age	0.012 (0.003)	<0.0001	0.017 (0.003)	<0.0001	0.014 (0.003)	<0.0001
Male gender	0.038 (0.061)	0.54	0.048 (0.063)	0.45	0.062 (0.062)	0.32
Random scanner effect						
SD of random effect (SE)	1.02 (0.07)		1.02 (0.06)		1.03 (0.05)	

Note that no test was performed for random effects.

## Discussion

The main findings of this study are that: 1) ADC histogram parameters appear highly correlated to MD values derived from DTI histograms previously used for assessing microstructural changes in cSVD, 2) image corrections such as CSF or artifacts removal have little effect on ADC measures over the whole brain, 3) conversely, scanner upgrades, as currently performed in a clinical setting over a large time scale, have major effects on measures derived from ADC histograms that can be even larger than the effects of age, sex or of the disease itself.

Metrics derived from whole brain MD histograms using DTI previously emerged as reliable and precise markers of disease severity and appeared particularly promising for monitoring disease progression in cSVD[10], [11], [13], [15], [21], [22].Although some studies based on routine DWI-derived ADC histograms already provided significant results[24], different diffusion MR techniques for assessing microstructural changes in cSVD have not been directly compared so far. In the present study, ADC histogram parameters obtained with DWI were found strongly correlated to parameters derived from MD histograms after CSF removal obtained with DTI and considered as the “gold standard” measure of diffusion in cSVD. In particular, the correlation was excellent when mean or height values of ADC histograms were compared to MD histogram values after CSF suppression. As might be expected, the concordance with the reference method was slightly altered in the absence of CSF suppression. These data strongly support that ADC histogram parameters from basic DWI as daily used in stroke centers may replace DTI measures for assessing tissue damage in cSVD.

Since the goal of using diffusion MR histograms is to globally quantify the microstructural brain tissue damage, considerable efforts are made to remove factors that may alter diffusion estimates such as the partial volume effect of increased CSF spaces in cortical atrophy or artifacts related to image distortion or those at the bone-air interface. Thus, various methods of CSF suppression have been proposed such as diffusion thresholding[14], [22], [24], fuzzy clustering-voxel based morphometry [15], [26] or the use of FLAIR-DWI[26], [30]. In this study we used a relatively low diffusivity threshold (18×10^−4^ mm^2^/s) after careful visual assessment of different threshold values (ranging from 16 to 28×10^−4^ mm^2^/s) to exclude voxels containing CSF before histogram generation. CSF removal had only slight effects on the correlation between ADC and MD histogram parameters. It did not change the peak location, but as expected, shifted the mean ADC to lower values and elevated the height of ADC histograms. Although moderate, the influence of CSF suppression was found to vary according to scanner upgrades. In contrast, removal of all bone-air artifacts manually or automatic suppression of the problematic top-bottom slices were found to have negligible effects on ADC histogram parameters in the cohort. The intra-class correlation coefficients were found always larger than 0.9 when ADC histograms were obtained before and after these interventions.

During this study, multiple upgrades of the MR scanner occurred as usually observed in a clinical setting over a large time scale. We showed in this study that these upgrades had major effects on diffusion measures. Such large variations were not initially expected since water diffusion as measured by MRI is mainly a physical characteristic of the tissue itself that should not be related to MR properties. However, significant effects related to the use of different scanners and/or imaging sequences on diffusion measures were previously reported in a small number of healthy volunteers[25]–[28]. These studies showed that ADC measures are significantly influenced by more or less important changes of hardware often performed over a long period in a clinical setting. The inter-scanner variability was previously found much greater than the inter-sequence variability but both were found relatively low in previous studies[25]. Growing maximum b-values were found to shift diffusion histograms to lower values, but scan-rescan results did not significantly differ[26]. In the present study, we observed that the variability related to the different scanner upgrades performed during the study period can far exceed the effects of the disease itself. In contrast, when the analysis was restricted to data obtained after the two last scanner upgrades which provided the most concordant diffusion data with the reference method, variations related to the scanner effect were found 10 times less than those related to patient characteristics. These data suggest that MR scanner upgrades can alter diffusion quantification during a long term follow-up study of cSVD but also that a reduction of related-variations can be obtained using appropriate technology and quality control as was proposed by a recent study on diffusion MRI of the breast[31]. The use of phantoms across upgrades for reducing scanner related variations may be particularly useful for correcting these variations.

In this study, the mean value appeared as the most useful parameter derived from whole brain ADC histograms for different reasons. The mean value is a continuous parameter and is thus more precise than the peak location which is necessarily discrete due to the use of bins for histograms. The mean value was also found to show the tightest correlation with the corresponding MD histogram parameter and was less influenced by the random scanner effect than the other parameters. Finally, the mean value of both ADC and MD histograms were previously reported to correlate with various clinical scores more than other histogram parameters both cross-sectionally and longitudinally[11], [15], [21], [22].

In conclusion, the results of the present study suggest that parameters of whole brain ADC histograms derived from clinically used DWI may be helpful in investigating cSVD in multicentric and large cohort studies. The mean ADC value measured over the whole brain after CSF suppression may represent a simple and relevant measure of microstructural changes in cSVD. However, the control of the major effects related to scanner upgrades on diffusion measures appears mandatory and the variability related to scanner or sequence changes on diffusion measures should be precisely estimated for long-term or multicentric studies.
